# Detection and complete genome characterization of a genogroup X (GX) sapovirus (family *Caliciviridae*) from a golden jackal (*Canis aureus*) in Hungary

**DOI:** 10.1007/s00705-024-06034-2

**Published:** 2024-04-17

**Authors:** Benigna Balázs, Ákos Boros, Péter Pankovics, Gábor Nagy, Sándor Szekeres, Péter Urbán, Gábor Reuter

**Affiliations:** 1 Department of Medical Microbiology and Immunology, Medical School, University of Pécs, Szigeti út 12., Pécs, H-7624 Hungary; 2 Department of Animal Physiology and Health, Hungarian University of Agriculture and Life Science, Kaposvár, Hungary; 3 Department of Parasitology and Zoology, University of Veterinary Medicine, Budapest, Hungary; 4 HUN-REN-UVMB Climate Change: New Blood-Sucking Parasites and Vector-Borne Pathogens Research Group, Budapest, Hungary; 5 Szentágothai Research Centre, Bioinformatics Research Group, Genomics and Bioinformatics Core Facility, University of Pécs, Pécs, Hungary

## Abstract

In this study, a novel genotype of genogroup X (GX) sapovirus (family *Caliciviridae*) was detected in the small intestinal contents of a golden jackal (*Canis aureus*) in Hungary and characterised by viral metagenomics and next-generation sequencing techniques. The complete genome of the detected strain, GX/Dömsöd/DOCA-11/2020/HUN (PP105600), is 7,128 nt in length. The ORF1- and ORF2-encoded viral proteins (NSP, VP1, and VP2) have 98%, 95%, and 88% amino acid sequence identity to the corresponding proteins of genogroup GX sapoviruses from domestic pigs, but the nucleic acid sequence identity values for their genes are significantly lower (83%, 77%, and 68%). During an RT-PCR-based epidemiological investigation of additional jackal and swine samples, no other GX strains were detected, but a GXI sapovirus strain, GXI/Tótfalu/WBTF-10/2012/HUN (PP105601), was identified in a faecal sample from a wild boar (*Sus scrofa*). We report the detection of members of two likely underdiagnosed groups of sapoviruses (GX and GXI) in a golden jackal and, serendipitously, in a wild boar in Europe.

The genus *Sapovirus* is one of the 11 genera of the family *Caliciviridae* [1]. Members of this genus have a positive-sense, single-stranded RNA genome with two or three open reading frames (ORFs). ORF1 encodes the non-structural polyprotein (NSP) and the major structural viral capsid protein (VP1). The predicted product of the ORF2 is a minor structural protein, VP2. ORF3 encodes a protein of approximately 160 amino acids (aa), but it is not present in some sapoviruses, including the porcine enteric calicivirus (PEC) Cowden strain [1, 2]. Both (5’ and 3’) genome ends contain short untranslated regions (UTRs). Sapoviruses can be divided into 20 genogroups (GI-GXX) based on the results of sequence comparisons and phylogenetic analysis based on the VP1 gene [1–3]. Sapoviruses are assigned to genogroups and genotypes based on nucleotide sequence comparisons of the complete VP1 region, with pairwise distance cutoff values of ≤ 0.488 and ≤ 0.169, respectively [2]. Viruses from four genogroups are known human pathogens, and these can be further subdivided into at least 17 genotypes [2].

Sapoviruses have been detected in a wide range of animals, including domestic pigs [4–7], chimpanzees [8], sea lions [9], minks [10], dogs [11], hyenas, lions, foxes [12], bats [13–15], rats [16], and wild boars [17], but not yet in golden jackals Members of four sapovirus genogroups (GI, GII, GIV, and GV) have been detected in humans, and members of nine (GIII, GV, GVI, GVII, GVII, GVIII, GIX, GX, and GXI) have been found in swine [18, 19]. However, most of the available scientific data are limited to certain sapovirus genogroups. Sapoviruses can cause mild-to-moderate acute gastroenteritis with diarrhoea in both humans and pigs [2].

The golden jackal (*Canis aureus*) is a mammal species of the dog family (Canidae) that is native to Southeast Europe, Central Asia, Western Asia, South Asia, and parts of Southeast Asia [20]. This species was almost extinct in Europe until the 1940s, except in Bulgaria; however, the golden jackal population increased in geographic distribution and abundance during the second half of the 20th century. Currently, there are an estimated 70,000 golden jackals in Europe, and they are present in many countries [21]. They are now listed as “Least Concern” on the IUCN Red List and can be hunted legally in Hungary [23]. Golden jackals and domestic dogs are able to produce fertile offspring [22]. Interactions between the growing golden jackal population and human livestock have become more common, and therefore, achieving a better understanding of potential pathogens spread by golden jackals is important.

Golden jackals can carry rabies virus [24], canine distemper virus [25], canine parvovirus [26], various endoparasites, and tick species [27, 28]. To our knowledge, there has been only one long-term study in which the presence of sapoviruses in golden jackals was investigated using molecular biology techniques, and no sapoviruses were found in that study [12].

In this study, we report the detection and complete genome characterization of a GX sapovirus obtained from the intestinal contents of a golden jackal. In addition, we detected a GXI sapovirus in faeces of a wild boar in Europe.

Two small-intestinal content specimens (DOCA-11 and DOCA-12) from golden jackals collected in 2020 in Pest County (near the village of Dömsöd), Hungary, were selected and pooled for viral metagenomic and next-generation sequencing (VM-NGS) analysis. Both animals were shot by professional hunters under the relevant hunting regulations [23]. The small intestines and their contents were dissected freshly and stored at -80^o^C until nucleic acid extraction. The sample preparation procedure for VM-NGS analysis was the same as described previously [29, 30]. Briefly, non-viral nucleic acids from the filtered (0.45 µm) enteric sample pool were digested with a nuclease cocktail of Turbo DNase (Ambion, Life Technologies, Grand Island, NY, USA), Baseline-ZERO (Epicentre, Chicago, IL, USA), and RNase One (Promega, Madison, WI, USA), and total nucleic acid was then isolated using a Quick-RNA Viral Kit (Zymo Research, Irvine, USA) according to the manufacturer’s instructions, but without the DNase-treatment step. After reverse transcription, cDNA and genomic DNA were amplified by random PCR. A library was constructed and sequenced on a NovaSeq 6000 (Illumina, San Diego, USA) platform [29, 30]. The resulting sequence data were analyzed using an in-house bioinformatics pipeline, using the RefSeq databases of NCBI, the Kaiju v1.7.3 and DIAMOND v2.1.6 aligners, and MEGAN6 v6.24.22 software for virus identification [29, 31–33].

The complete sapovirus genome sequence was determined by a combination of RT-PCR techniques, including 3’/5’ RACE PCR, long-range PCR, and primer-walking methods, using protocols that have been described elsewhere [29, 30, 34, 35]. The oligonucleotide primers used for determination of the complete genome sequence were designed based on the aligned sapovirus metagenomic reads and on aligned sequences of the most closely related GX genogroup sapovirus strains identified using BLAST searches. PCR products were sequenced by the dye-terminator sequencing method, using a BigDye Terminator v1.1 Cycle Sequencing Kit (Thermo Fisher Scientific) and an automated sequencer (AB3500 Genetic Analyzer, Applied Biosystems, Hitachi, Tokyo, Japan).

Amino acid sequence alignments of the non-structural proteins (NSP) and capsid VP1 proteins of the strains from this study and representative prototype sapovirus strains available in the GenBank database were made using GeneDoc (version 2.7) and the MUSCLE web server. Phylogenetic analysis was performed using MEGA X (version 10.2.3), using the neighbor-joining method and the Jones-Taylor-Thornton model with 1000 bootstrap replications [36]. RNA secondary structures in the 5′ end/coding region and the junction of the non-structural protein/structural protein regions were predicted using Mfold software [37] and visualized using CorelDraw Graphics Suite v. 12.

A primer pair (F: 5’-CCA TCA GGG ATG CCA GGC A-3’) corresponding to nt 4354–4372 of the study strain and R: 5’-CCC TCC ATC ACA TAC ACT ATT-3’, corresponding to nt 4979 − 4958) was designed for sapovirus screening based on the aligned sapovirus sequences of the study strain GX/Dömsöd/DOCA-11/2020/HUN (PP105600) and strain GX/HgTa2/2016 (LC215896.2) [38], its closest relative. This primer pair was used for further screening of additional faecal and small-intestinal content specimens collected from other golden jackals that had also been shot by professional hunters (Table 1), faecal specimens from wild boars collected near Tótfalu (Vas County), and domestic pigs collected from four farms in Orosháza (Békés County), Városföld, Katymár, and Bácsalmás (Bács-Kiskun County) in Hungary. Viral RNA was isolated from the faecal and intestinal content samples using TRIzol Reagent (Thermo Fisher Scientific, Waltham, MA, USA) according to the manufacturer’s instructions. Conventional RT-PCR was performed using the sapovirus screening primer pair with a PCR program consisting of an initial denaturation of 30 s at 95ºC, 40 cycles of 35 s at 95ºC, 20 s at 57ºC, and 1 min at 72ºC, and a final 5-minute elongation step at 72ºC, using a C1000 Touch Thermal Cycler (Bio-Rad).

**Table 1:** Detailed background information about the golden jackals and samples used for the epidemiological investigation of sapovirus GX as well as the results of the RT-PCR test

Sample ID	Date of sampling	Geographical location of sampling (long./lat.)	County	Sample type	Sex	Age (months)	Result of sapovirus screening RT- PCR
RG1	15. 04. 2023	46.197834/17.800639	Somogy	Faeces	n.a	n.a	Negative
RG2	15. 04. 2023	46.201339/17.803166	Somogy	Faeces	n.a	n.a	Negative
RG3	15. 04. 2023	46.222987/17.796335	Somogy	Faeces	n.a	n.a	Negative
LB168	19. 01. 2023	46.736479/17.874728	Somogy	Small intestinal contents	Male	24	Negative
TK396	16. 01. 2023	45.796106/17.843309	Somogy	Small intestinal contents	Female	12>	Negative
TK397	16. 01. 2023	45.796106/17.843309	Somogy	Small intestinal contents	Female	12>	Negative
TK398	16. 01. 2023	45.796106/17.843309	Somogy	Small intestinal contents	Female	20	Negative
TK399	16. 01. 2023	45.796106/17.843309	Somogy	Small intestinal contents	Male	20	Negative
TK400	16. 01. 2023	45.796106/17.843309	Somogy	Small intestinal contents	Female	100	Negative
TK401	16. 01. 2023	45.796106/17.843309	Somogy	Small intestinal contents	Male	44	Negative
TK402	16. 01. 2023	45.796106/17.843309	Somogy	Small intestinal contents	Male	32	Negative
TK414	06. 02. 2023	46.269023/17.652537	Somogy	Small intestinal contents	Male	34	Negative
TK415	11. 02. 2023	46.26902/17.65254	Somogy	Small intestinal contents	Male	20	Negative
TK417	11. 02. 2023	46.069193/17.632049	Somogy	Small intestinal contents	Male	20	Negative
TK418	11. 02. 2023	46.069194/17.632050	Somogy	Small intestinal contents	Male	20	Negative
TK419	11. 02. 2023	46.069195/17.632051	Somogy	Small intestinal contents	Male	20	Negative
TK420	11. 02. 2023	46.069196/17.632052	Somogy	Small intestinal contents	Male	< 12	Negative
TK421	11. 02. 2023	46.069197/17.632053	Somogy	Small intestinal contents	Male	< 12	Negative
TK423	12. 02. 2023	46.069198/17.632054	Somogy	Small intestinal contents	Female	60	Negative
TK434	13. 02. 2023	46.18721/17.76497	Somogy	Small intestinal contents	Male	60	Negative
TK437	14. 02. 2023	46.269028/17.652499	Somogy	Small intestinal contents	Female	60	Negative
TK439	11. 04. 2023	46.831/17.9983	Somogy	Small intestinal contents	Female	24	Negative
TK440	11. 04. 2023	46.831/17.9983	Somogy	Small intestinal contents	Male	24	Negative
TK442	13. 04. 2023	46.291168/17.634222	Somogy	Small intestinal contents	Male	12	Negative
TK444	14. 04. 2023	46.290900/17.633178	Somogy	Small intestinal contents	Male	12<	Negative
TK448	15. 04. 2023	46.291536/17.633816	Somogy	Small intestinal contents	Male	12>	Negative
Dömsöd/DOCA-11	19. 05. 2020	47.077919/19.028257	Pest	Small intestinal contents	Female	12<	**Positive**
Dömsöd/DOCA-12	19. 05. 2020	47.077919/19.028257	Pest	Small intestinal contents	Male	12<	Negative

Using viral metagenomics and next-generation sequencing methods, a total of 33,102,106 paired reads, including 72,128 viral reads were obtained from the small-intestine content samples of the two golden jackals. Of these, 41,322 were identified as belonging to members of the family *Partitiviridae*, 21,914 to the family *Picobirnaviridae*, 8,730 to the family *Spinareoviridae*, 62 to the family *Autographiviridae*, 32 to the family *Straboviridae*, 18 to the family *Endornaviridae*, 16 to the family *Totiviridae*, 10 to the family *Retroviridae*, eight to the family *Mimiviridae*, eight to the family *Astroviridae*, and eight to the family *Caliciviridae* (all of which were sapoviruses). Sapovirus sequence reads were selected for further investigation. The presence of a sapovirus was confirmed in one (Dömsöd/DOCA-11) of the two specimens by RT-PCR and Sanger sequencing, using read-specific oligonucleotide primers.

The complete genome length of the sapovirus strain GX/Dömsöd/DOCA-11/2020/HUN (PP105600) is 7,128 nt excluding the poly(A) tail. ORF1, comprising the NSP and VP1 coding regions, is 6,579 nt long and encodes a 2,192-aa-long polyprotein. This polyprotein starts with an MVATCHHSIC sequence, which is consistent with the conserved MxAxCxHxxC consensus sequence found in GX sapoviruses [39]. The non-structural protein coding region had 83% nt and 98% aa sequence identity to the closest relative, GX/HgTa2/2016 (LC215896.2), from a domestic pig [38], identified in the database using GenBank BLASTn and BLASTx, respectively. The three conserved aa motifs in the putative NTPase (GXPGXGKT, WDE(F/Y)D, and PL(N/D)CD) and the two aa motifs n the VPg (KGKXX and XDEYXX) described by Kuroda et al. [38] were recognisable in the study strain (G_453_PPGIGKT, W_495_DEYD, P_520_LNCD, K_920_GKNK, and DD_933_EYTE) and are identical to those in strain GX/HgTa2/2016. The VP1 coding region is 1,617 nt long, encoding a 538-aa-long capsid protein. This region has 77% nt and 95% aa sequence identity to the two most closely related sapovirus strains, GX/HgTa2/2016 and K8/JP (AB242873), respectively [38, 40]. The VP2-coding region is 507 nt in length, encoding a 168-aa-long minor capsid protein. This region has 73% nt and 88% aa sequence identity to the corresponding region of the strain HgTa3-2/2016 (LC215897.2) [38], which is higher than the nt/aa identity (68% nt and 82% aa) to the corresponding region of strain GX/HgTa2/2016.

Based on the results of sequence comparisons and phylogenetic analyses of the non-structural proteins and VP1 capsid proteins, we conclude that strain GX/Dömsöd/DOCA-11/2020/HUN (PP105600) belongs to genogroup GX (Fig. 1) and potentially represents a novel sapovirus genotype, which we have tentatively named "GX.3" (Fig. 1B).

**Fig. 1:**
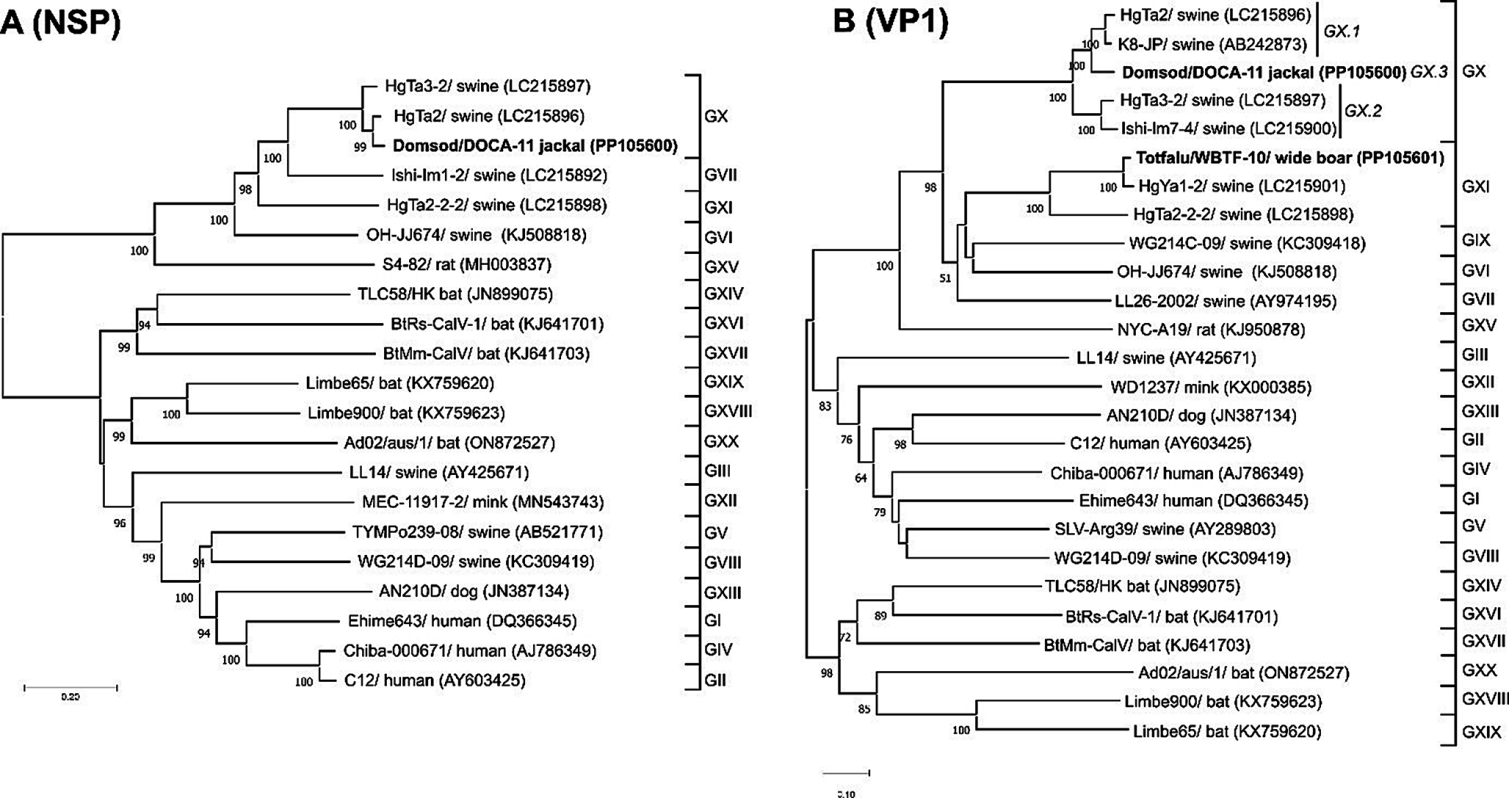
Phylogenetic analysis based on the non-structural proteins (**A**) and VP1 capsid proteins (**B**) of the two study strains (in bold) and representatives of different sapovirus genogroups (GI-GXX). Strain GX/Dömsöd/DOCA-11/2020/HUN (PP105600) from golden jackal is shown on both trees, strain GXI/Tótfalu/WBTF-10/2012/HUN (PP105601) from wild boar is shown only on the VP1 tree due to the lack of a complete NSP sequence. The trees were generated from MUSCLE-based amino acid (aa) sequence alignments by the neighbor-joining method, with the Jones-Taylor-Thornton model with 1000 bootstrap replications in MEGA X. Only bootstrap values higher than 50 are shown. The trees are drawn to scale, with branch lengths indicating the number of aa substitutions per site. The sequence designations are as follows: sapovirus strain name/host and accession number. Note that the only available partial non-structural protein sequence for genogroup IX (KC309418) was excluded from the analysis of NSP sequences

The 5’UTR of strain GX/Dömsöd/DOCA-11/2020/HUN (PP105600) is 9 nt in length and is 100% identical to the 5’UTR of strain GX/HgTa3-2/2016. The 3’UTR is 37 nt in length, excluding the poly(A) tail, and no similar sequence was found in the GenBank database. We predicted the secondary RNA structure of the 5’UTR/coding region and the NSP/VP1 junction genome regions (Fig. 2), which were found to have the closest structural similarity to the corresponding structures of strain GX/HgTa3-2/2016 [38].

**Fig. 2:**
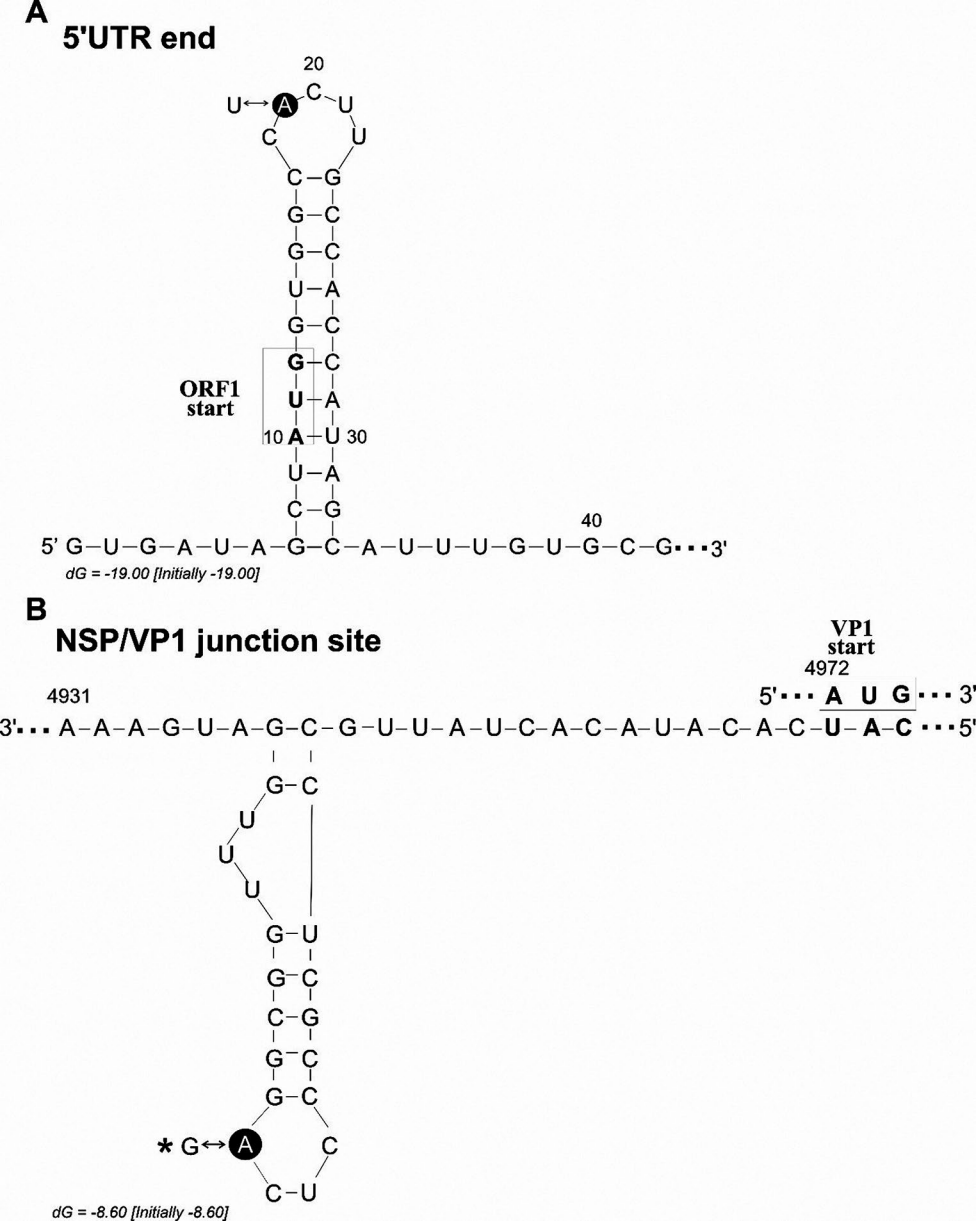
(**A**) The predicted secondary RNA structure of the 5’UTR sequence of strain GX/Dömsöd/DOCA-11/2020/HUN (PP105600). The first 42 nt were used to predict the structure, using Mfold [37]. The star marks the position (A – U) where the sequence differs from that of strain GX/HgTa3-2/2016 [39]. Please note that modifications have been made to the GX/HgTa3-2/2016 and GX/HgTa2/2016 sequences since their publication [39]. We used the corrected data for comparison between the GX/Dömsöd/DOCA-11/2020/HUN strain and these strains. Nucleotides in boldface type in the frame with the arrow represent the start codon of ORF1. (**B**) The predicted secondary RNA structure of the complementary (antisense) strand of the NSP/VP1 junction site shown in the 3’-to-5’ direction. The star marks the single nt difference (A – G) compared to strain GX/HgTa2-3/2016 [39]. Nucleotides in boldface type in the frame represent the start codon of VP1

Until now, GX sapoviruses have been detected only in domestic pigs. We further investigated the presence of GX sapovirus in additional enteric samples from 23 golden jackals (Table 1) and in faecal samples from 16 domestic pigs and 10 wild boars, by the RT-PCR method, using the newly designed sapovirus screening primer pair. None of the golden jackal or domestic pig specimens were positive, but one of the 10 faecal samples (WBTF-10) from wild boar was positive and confirmed by Sanger sequencing to contain a sapovirus (GXI/Tótfalu/WBTF-10/2012/HUN, PP105601) (Fig. 1B). After determining the 4,230-nt-long 3’ part of the genome, including the region encoding the capsid protein, a comparison of VP1 sequences showed that it had a high degree of similarity (97% aa and 82% nt sequence identity) and a close phylogenetic relationship (Fig. 1B) to the corresponding protein of GXI sapovirus strain GXI/HgYa1-2/2016 (LC215901), which was identified in a faecal sample from a domestic pig in Japan [38].

In this study, we identified and characterized a novel sapovirus strain from the small-intestinal contents of a golden jackal. This is the first report of the detection of a sapovirus in a golden jackal. The newly identified strain belongs to genotype GX. Members of this genogroup were detected previously only in faecal samples from domestic pigs in Japan [38, 40] and Europe [41, 42].

There are only two available complete genome sequences of GX sapovirus [38, 39]. These sequences suggest that GX sapoviruses have a shorter genome than do members of other genogroups (except GVI and GVII) and that they share some common features, including (i) a shorter ORF1, (ii) a common first 10 amino acid residues (MxAxCxHxxC) of the ORF1 protein, and (iii) common predicted RNA secondary structures of the 5’UTR and the junction of the non-structural protein/structural protein regions [39].

The VP1 protein of strain GX/Dömsöd/DOCA-11/2020/HUN shows 95% aa sequence identity to the corresponding proteins of strains GX/HgTa2/2016 and K8/JP; however, the nt sequence identity in the VP1 coding region was significantly lower (only 77%), indicating a large number of synonymous nucleotide variations. Based on the sapovirus genotype cutoff criteria (< 0.169) [2], strain GX/Dömsöd/DOCA-11/2020/HUN may represent a novel genotype (GX.3) within GX. Sapoviruses, including GX are probably genetically much more diverse. Based on the results of sequence comparisons, the strain from this study could not be detected using the widely used generic calicivirus screening primer pair p289/p290, which was designed for a wide range of noro- and sapoviruses [43], because of the multiple primer mismatches. Presumably, the primers that are currently used for epidemiological studies do not cover the whole diversity of sapovirus variants, and underreporting of infections is therefore likely.

There have been only a limited number of studies reporting the presence of GX sapoviruses, and this genogroup has only been found in domestic pigs. Kuroda et al. tested 105 pig faeces and found three sapovirus GX strains, indicating a prevalence of 2.9% among their studied samples [38]. In a pan-European study, 14 out of 1,050 faecal samples (1.3%) were found to be positive for GX sapovirus. Positive samples were collected from Denmark (N = 10), Finland (N = 2), Italy (N = 1), and Slovenia (N = 1), but not from Hungary [41]. Another study from Italy found six (2%) GX sapovirus cases out of the 290 tested faecal specimens [42]. These results indicate a relatively low detection rate of sapovirus GX strains in pigs.

There are many carnivorous animals in which sapoviruses have been detected, including minks, lions, sea lions, dogs, hyenas, and foxes [9–12]. The sapoviruses found in African carnivores were different from those found in their prey [12]. Dogs and some studied minks had diarrheic symptoms, and their sapoviruses also differed significantly from those found in other species [10, 11]. Based on these findings, carnivorous animals, including canids related to golden jackals, could be the natural hosts of sapoviruses, but they could also carry sapoviruses of dietary origin. To address this possibility, we carried out screening tests on faecal samples from domestic pigs and wild boars, but no GX sapoviruses were identified in the specimens collected in Hungary. However, we serendipitously detected a sapovirus GXI strain in a faecal sample from a wild boar in Europe. Until now, only GIII, GV, and GVI sapoviruses had been reported in wild boars [17], and GXI sapoviruses have been identified in only five cases in domestic pigs in Japan and Canada [38, 39, 44]. A sequence analysis of the VP1 protein suggests that strain GXI/Tótfalu/WBTF-10/2012/HUN may represent a novel genotype within genogroup GXI. This result indicates a wider host spectrum (wild boar in addition to domestic pig) and a wider geographic distribution of this sapovirus genogroup.

In conclusion, we report the detection and complete genome characterization of a possibly underdiagnosed group(s) of sapoviruses (GX and GXI) from enteric samples from a golden jackal and a wild boar in Europe. This study extends our knowledge about the genome sequence and host species diversity and the geographical distribution of certain types of sapoviruses.

## Data Availability

The nucleotide sequence data reported here are available in the DDBJ/EMBL/GenBank databases under the accession numbers PP105600 and PP105601.
